# Supplementary material for: Senescence-associated 13-HODE production promotes age-related liver steatosis by directly inhibiting catalase activity
Source: Nat Commun. 2023 Dec 9;14:8151. doi: 10.1038/s41467-023-44026-z (PMC10710422; doi:10.1038/s41467-023-44026-z)
Supplement: Supplementary file 1 — Supplementary Information [file 41467_2023_44026_MOESM1_ESM.pdf]

Senescence-associated 13-HODE production promotes age-related liver steatosis by directly inhibiting catalase activity

Supplemental data

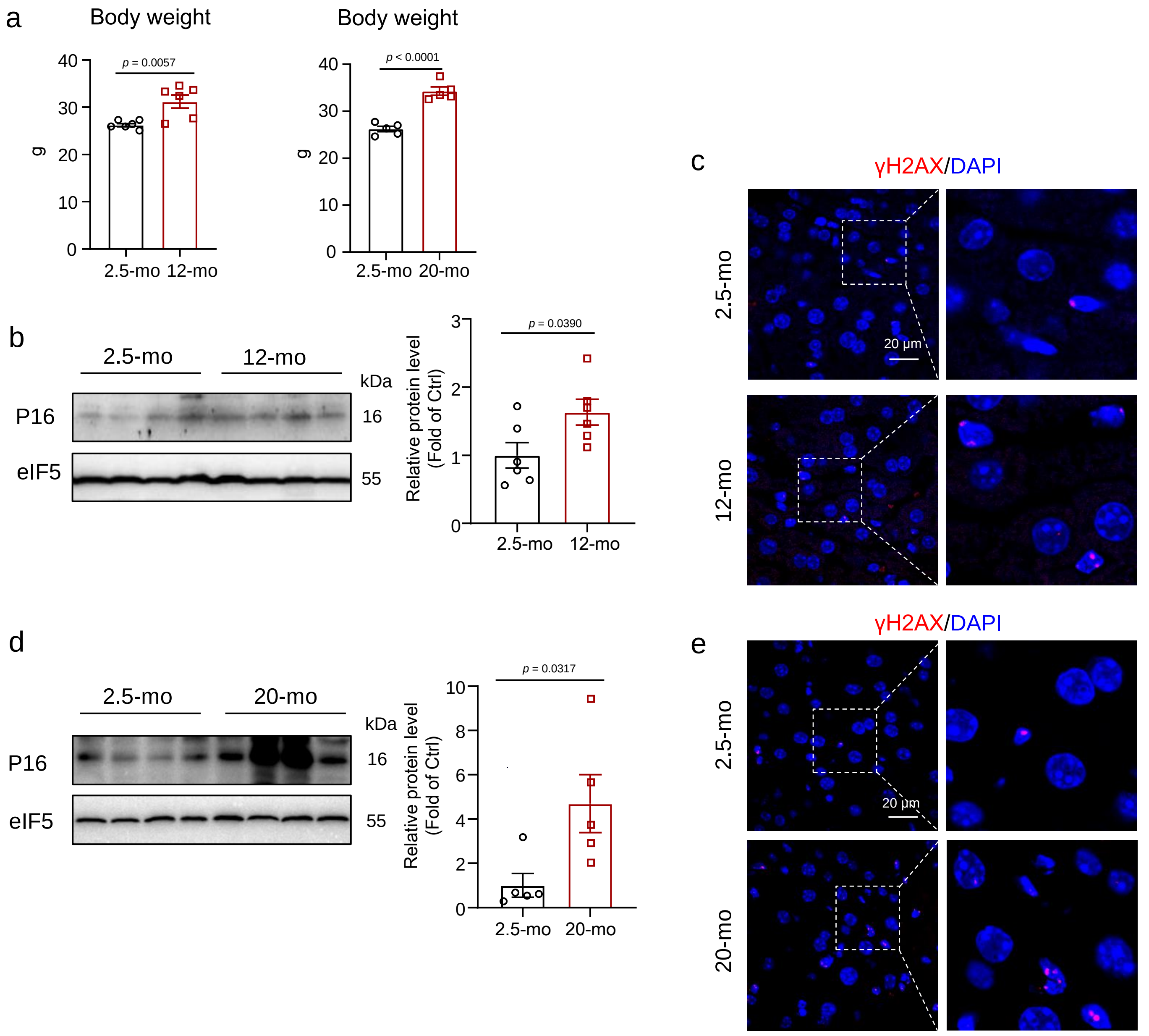

Fig. S1 Senescent markers of middle-aged and aged mouse livers.
(a) Body weight of mice at age 12 months (middle-aged, n = 6 mice per group) or 20 months (aged, n = 5 mice per group). (**b–e**) western blot analysis of the protein level of P16 and immunofluorescence staining of γH2AX in livers from mice aged 2.5 months, 12 months, or 20 months; scale bar = 20 μm. Data represent the mean ± SEM. Two-tailed student's t test was performed for (a-b); Two-tailed Mann-Whitney test was performed for (d). mo: month.

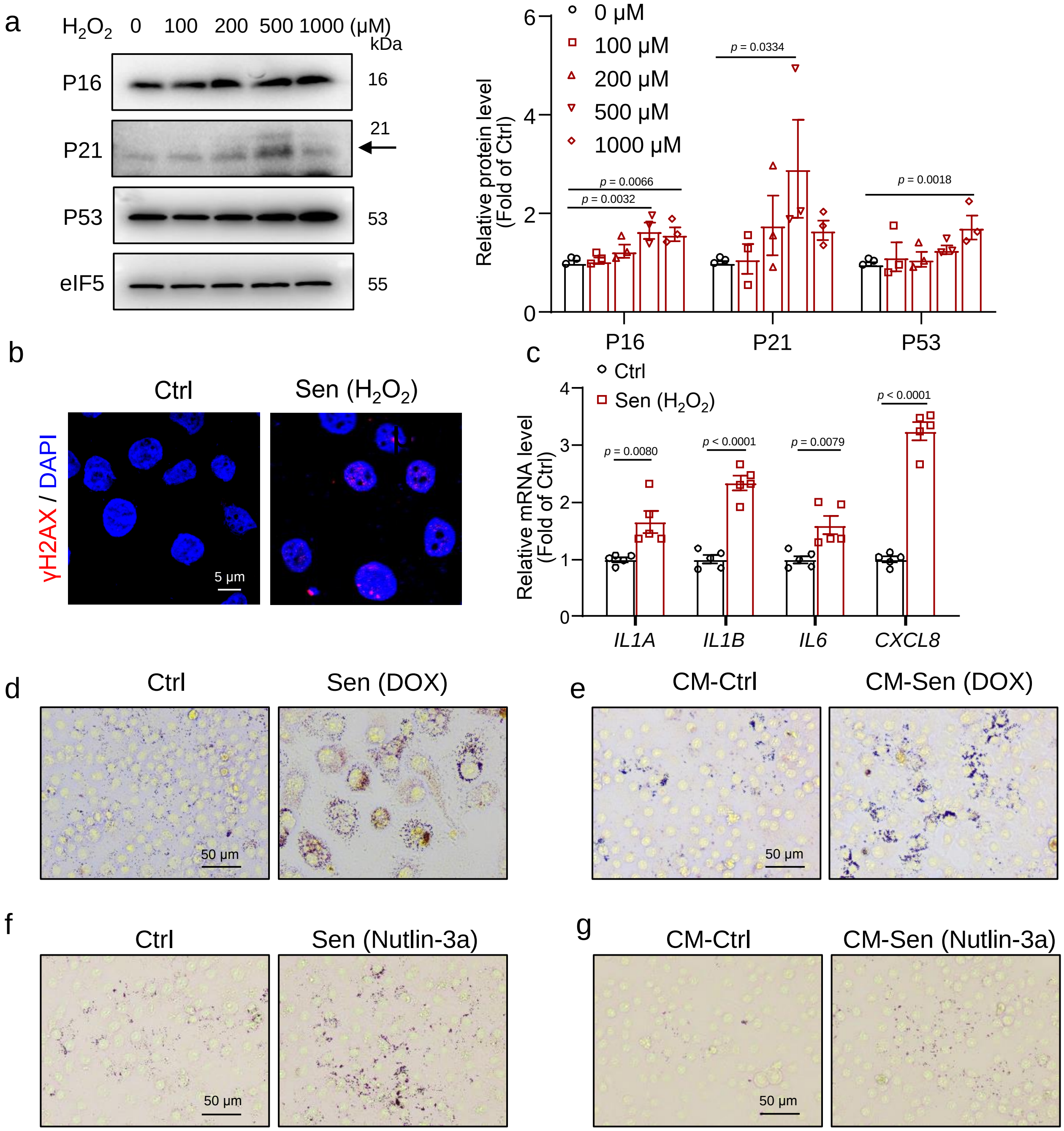

Fig. S2 Senescent hepatocyte models.

(a–c) Markers of senescence in H_2O_2-induced senescent hepatocytes. (a) HepG2 cells were treated with H_2O_2 at the indicated concentration for 48 h, and western blot analysis of the protein levels of P16, P21 and P53. (b) Immunofluorescence staining of γH2AX; scale bar = 5 μm. (c) qPCR analysis of mRNA levels of *IL1A*, *IL1B*, *IL6*, and *CXCL8*. (a, b) n = 3 independent experiments, (c) n = 5 independent experiments. **(d–g)** HepG2 cells were treated with 2 μM Doxorubicin (DOX) for 2 h and then cultured in DOX-free medium for 6 days to induce senescence (d, e) or Nutlin-3a (10 μM; f, g) for 24 h to induce senescence. Oil Red O staining of senescent hepatocytes or control hepatocytes (d, f); HepG2 cells were treated with conditioned medium from senescent hepatocytes or control hepatocytes for 24 h, and then Oil Red O staining was performed (e, g); n = 5 independent experiments; scale bar = 50 μm. Data represent the mean ± SEM. One-way ANOVA with Fisher's LSD was performed for (a); Two-tailed student's t test was performed for (*IL1A, IL1B and CXCL8* in c); Two-tailed Mann-Whitney test was performed for (*IL6* in c). CM: conditioned medium; Sen: senescent.

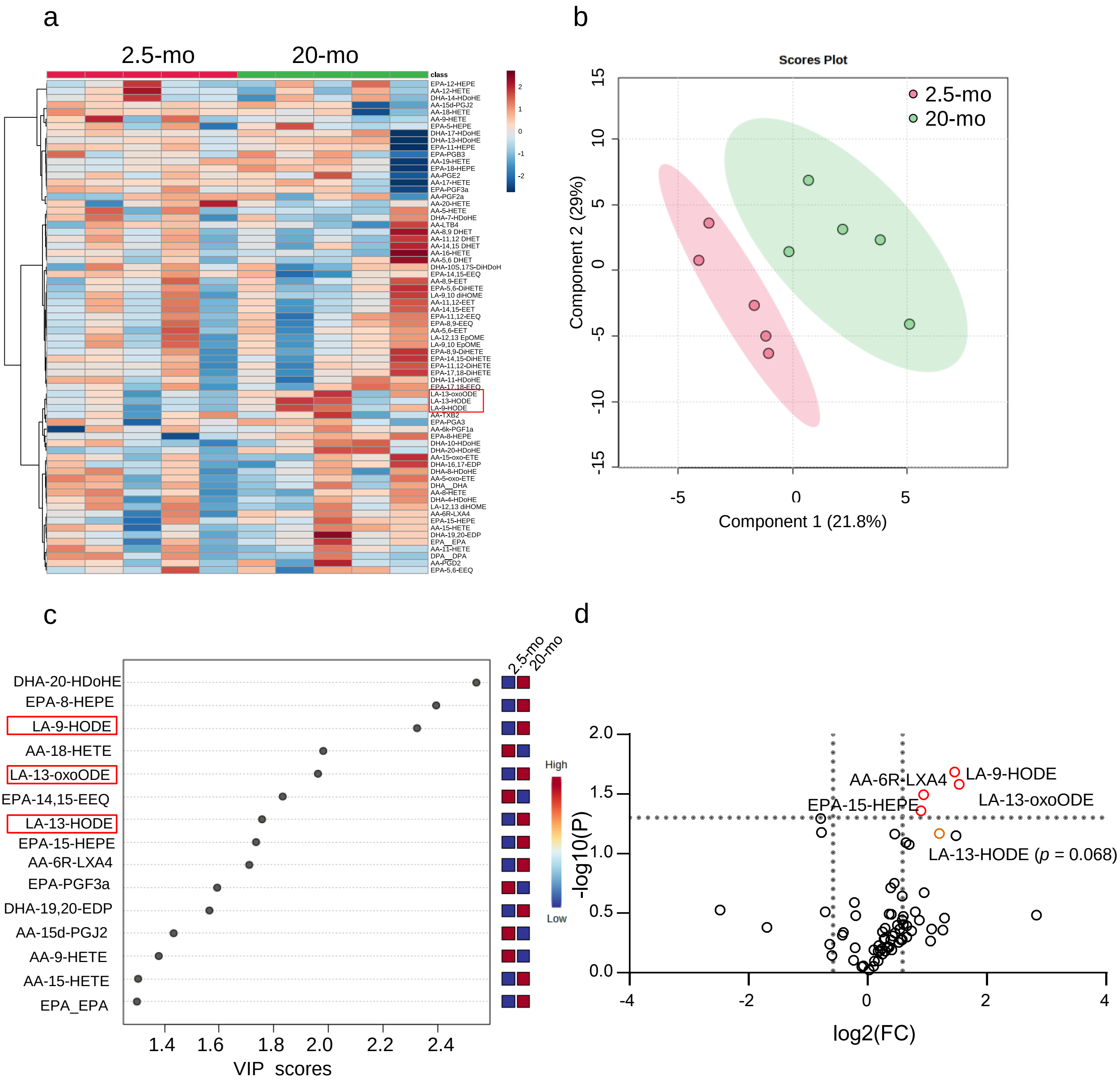

Fig. S3 PUFA metabolite profile of livers of aged mice.
Liquid chromatography–tandem mass spectrometry (LC–MS/MS) was performed to detect the PUFA metabolite profile of 2.5- and 20-month-old mice: **(a)** heatmap; **(b)** PLS-DA analysis, **(c)** VIP scores and **(d)** volcano plot of significantly changed metabolites. n = 5 mice per group. mo: month; AA: Arachidonic acid; LA: linoleic acid; EPA: eicosapentaenoic acid.

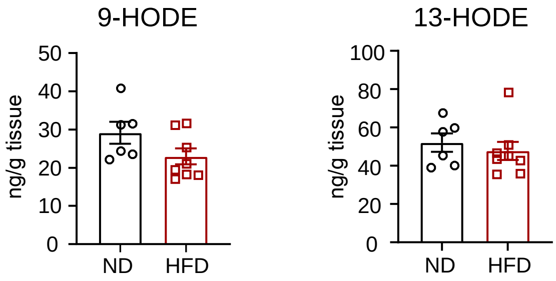

Fig. S4 Levels of 9-HODE and 13-HODE in normal and high fat diet fed mouse livers.
Eight-week-old mice were fed with normal or high fat diet for 12 weeks. Levels of 9-HODE and 13-HODE in mouse livers were measured by LC–MS/MS. n = 6 mice in Ctrl group; n = 8 mice in HFD group. Data represent the mean ± SEM. Two-tailed student’s t test was performed for 9-HODE and Two-tailed Mann Whitney test was performed for 13-HODE. ND: normal diet; HFD: high fat diet.

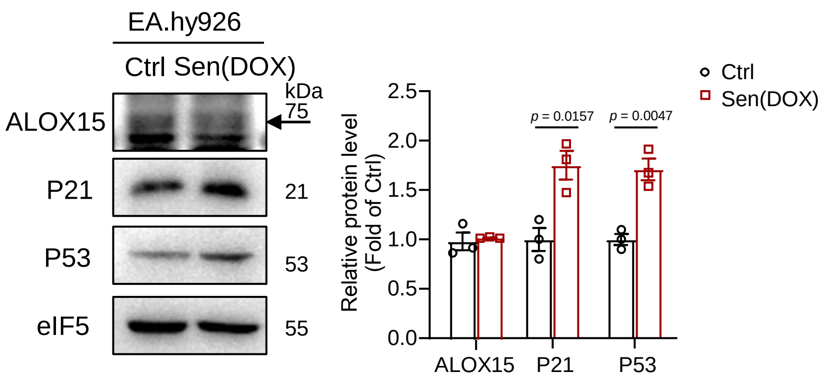

Fig. S5 ALOX15 expression was unchanged in senescent endothelial cells. EA.hy926 cells were treated with 0.5 μM DOX for 2 h and then cultured in DOX-free medium for another 2 days to induce senescence: western blot analysis of the protein levels of ALOX15, P21, and P53. n = 3 independent experiments. Data represent the mean ± SEM. Two-tailed student's t test was performed.

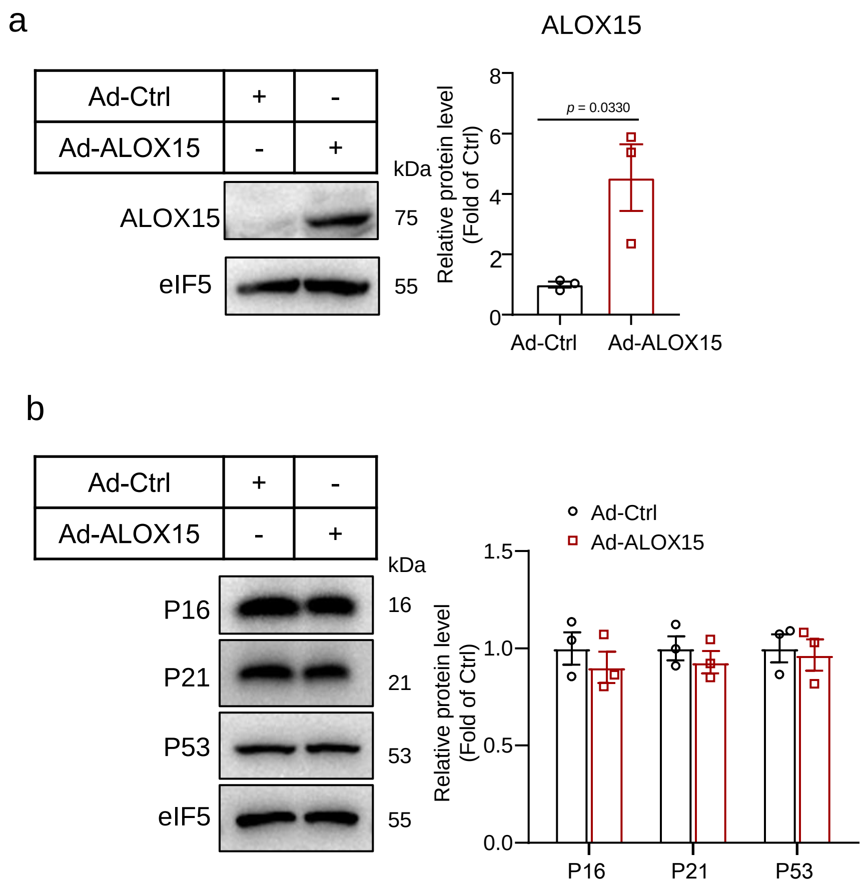

Fig. S6 ALOX15 overexpression did not affect senescent markers in hepatocyte.
Primary mouse hepatocytes were infected with Ad-ALOX15 or Ad-Ctrl for 48 h: western blot analysis of the protein levels of ALOX15 (a), P16, P21, and P53 (b). n = 3 independent experiments. Data represent the mean ± SEM. Two-tailed student's t test was performed for (a-b).

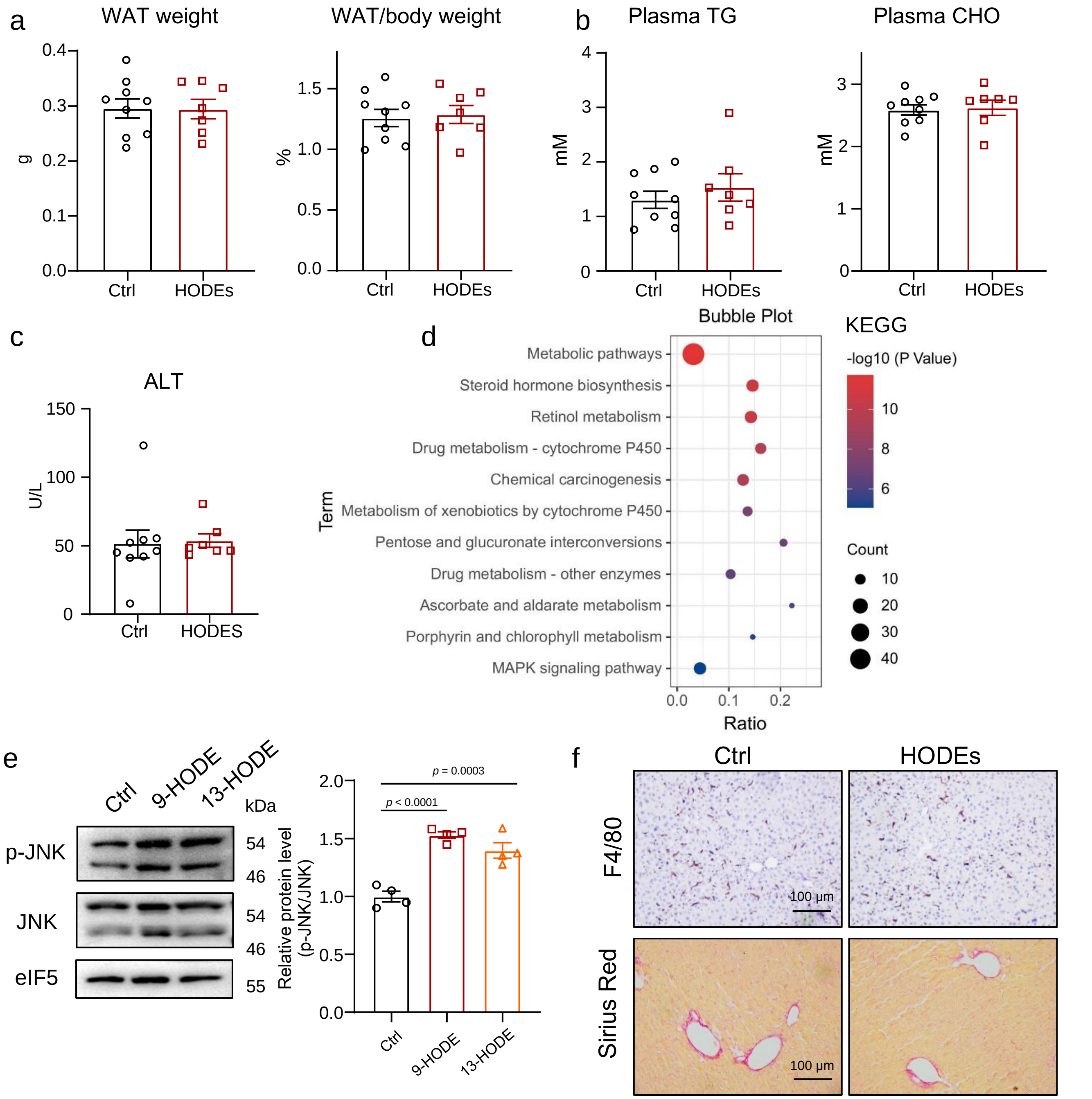

Fig. S7 Effects of 9-HODE and 13-HODE on MAPK signaling.

Eight-week-old mice were treated with a mixture of 9-HODE and 13-HODE (equal amounts of 9-HODE and 13-HODE and a combined dose of 0.5 μg/g body weight) once a day for 9 days (n = 9 mice in Ctrl group; n = 7 mice in HODE group): **(a)** epididymal white adipose tissue (eWAT) and eWAT/body weight; **(b)** plasma levels of TG and CHO; **(c)** plasma level of ALT; **(d)** KEGG enrichment of differentially expressed genes. **(e)** Primary mouse hepatocytes treated with 9-HODE or 13-HODE (1 Mm) for 48 h: western blot analysis of the protein levels of p-JNK and JNK; n = 4 independent experiments. **(f)** Immunohistochemical staining of F4/80 and Sirius red staining of liver sections of these mice, scale bar = 50 μm. Data represent the mean ± SEM. Two-tailed student's t test was performed for (a-b); Two-tailed Mann-Whitney test was performed for (c); One-way ANOVA with Fisher's LSD was performed for (e). TG: triglyceride; CHO: total cholesterol; ALT: Alanine aminotransferase; HODEs: 9/13-HODEs.

a

	Average FPKM Ctrl	Average FPKM HODEs	p-value
G6pc	37.94	60.18	0.002

b

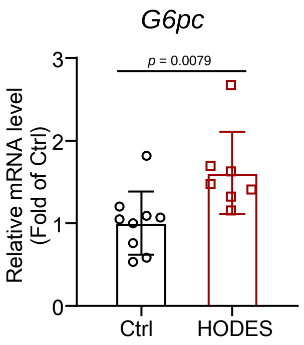

c

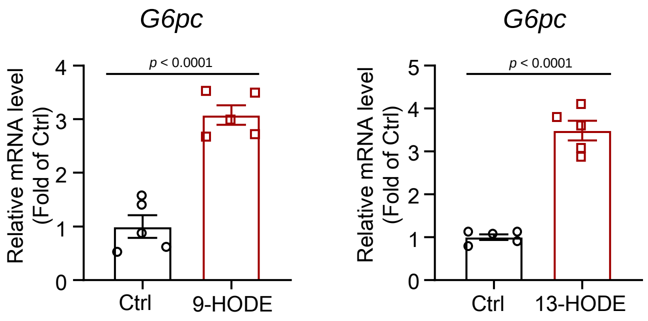

d

	Average FPKM Ctrl	Average FPKM HODEs	p-value
Trp53	4.29	3.79	0.68
Cdkn1a	0.32	0.18	0.09
Cdkn2a	0.05	0.06	1

e

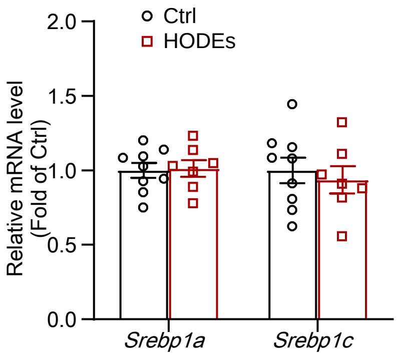

Fig. S8 Expression levels of *G6pc*, *Srebp1a*, *Srebp1c*, and senescent markers in livers of mice treated with a combination of 9-HODE and 13-HODE.

Eight-week-old mice were treated with a mixture of 9-HODE and 13-HODE (equal amounts of 9-HODE and 13-HODE and a combined dose of 0.5 µg/g body weight) once a day for 9 days: **(a)** expression level of *G6pc* from RNA sequencing; **(b)** qPCR analysis of mRNA level of *G6pc*; n = 9 mice in Ctrl group; n = 7 mice in HODE group. **(c)** Primary mouse hepatocytes treated with 9-HODE or 13-HODE (1 µM) for 48 h: qPCR analysis of the mRNA level of *G6pc*; n = 5 independent experiments. **(d)** Expression levels of *Trp53*, *Cdkn1a*, and *Cdkn2a* from RNA sequencing. **(e)** qPCR analysis of mRNA level of *Srebp1a* and *Srebp1c* in livers of these mice; n = 9 mice in Ctrl group; n = 7 mice in HODE group. Data represent the mean ± SEM. Two-tailed Mann-Whitney test was performed for (b). Two-tailed student's t test was performed for (c) and (e). HODEs: 9/13-HODEs.

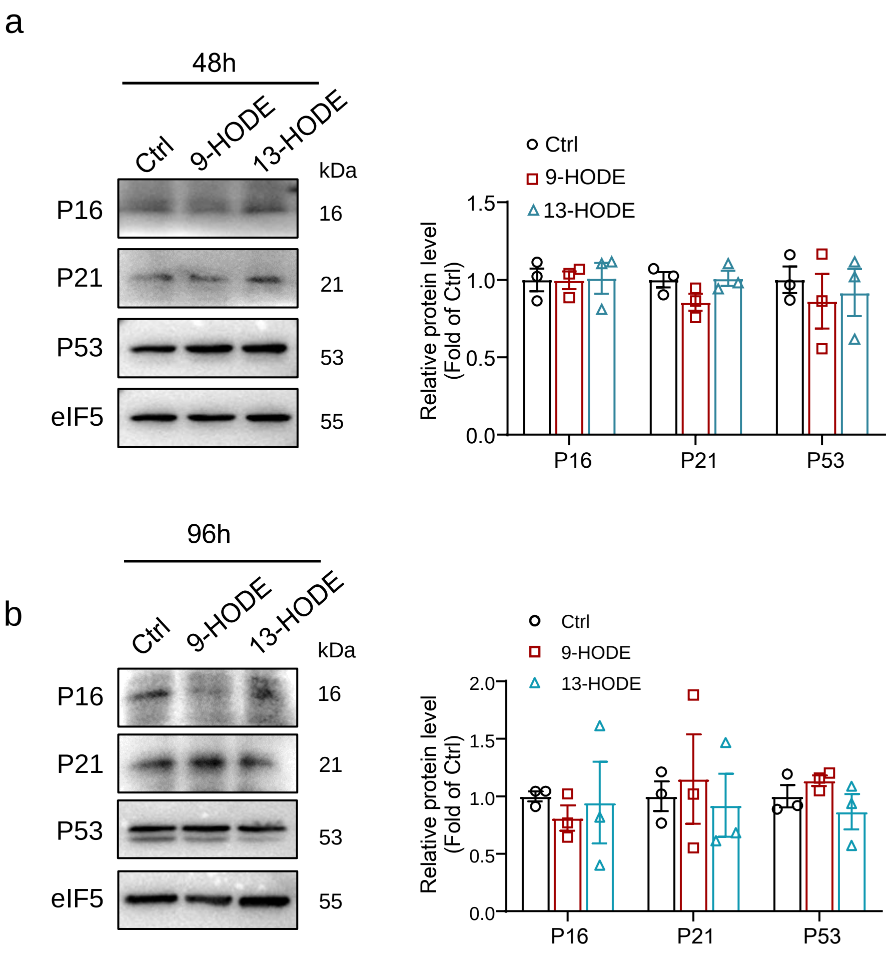

Fig. S9 Effects of 9-HODE and 13-HODE on hepatocyte senescence.
Primary mouse hepatocytes treated with 9-HODE or 13-HODE (1 μM) for 48 h **(a)** or 96 h **(b)**: western blot analysis of the protein levels of P16, P21, and P53; n = 3 independent experiments. Data represent the mean ± SEM. One-way ANOVA with Fisher's LSD was performed for (a-b).

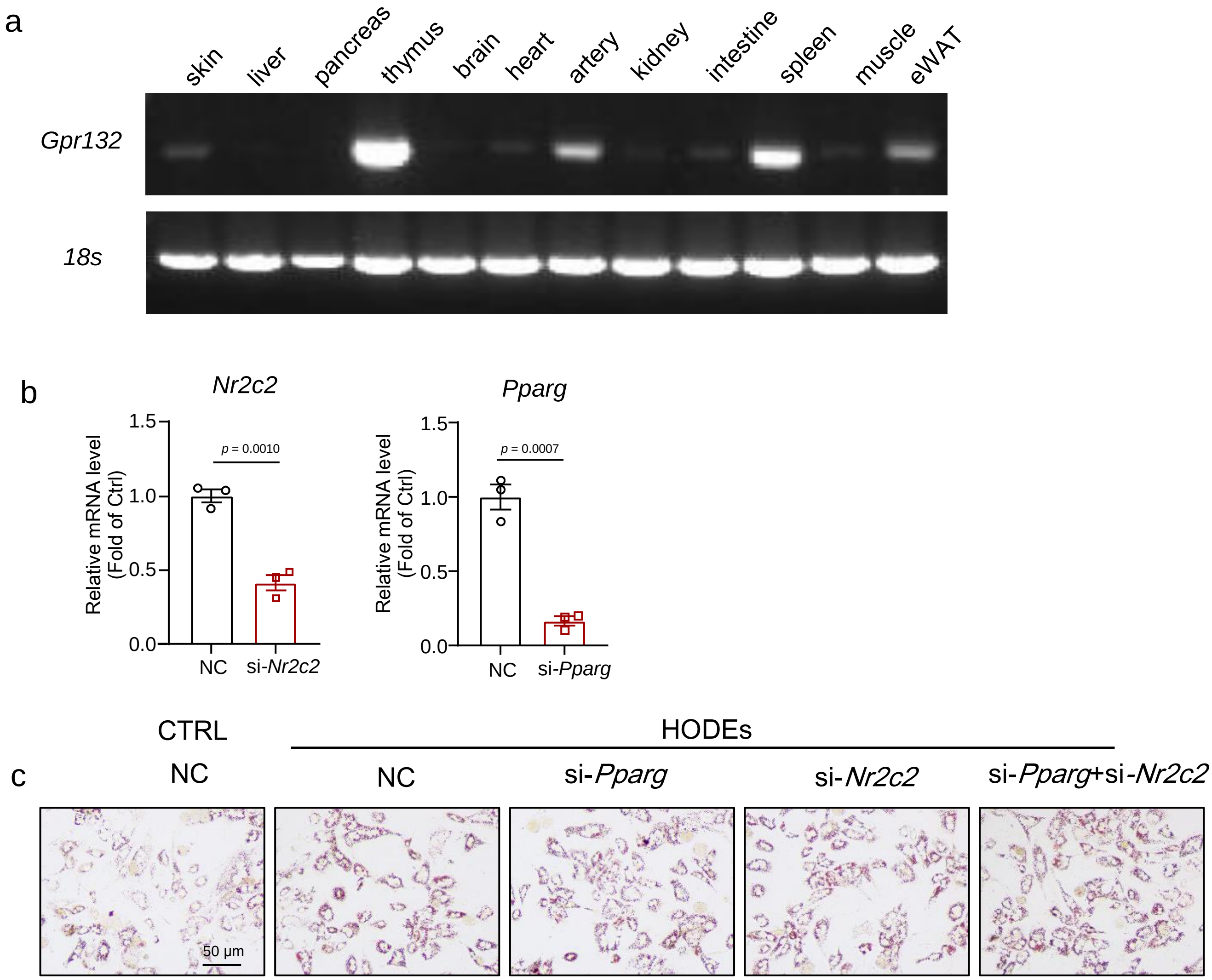

Fig. S10 Expression of *Gpr132* in various tissues and the effects of NR2C2 and PPARγ on hepatocyte steatosis induced by the mixture of 9-HODE and 13-HODE.

(a) Expression of *Gpr132* in the indicated tissues detected by RT–PCR followed by agarose gel electrophoresis. **(b, c)** Primary mouse hepatocytes were transfected with si-*Nr2c2*, si-*Pparg*, or both with or without treated with a mixture of 9-HODE and 13-HODE (equal amounts of 9-HODE and 13-HODE with a total concentration of 1 μM) for 48 h. (b) qPCR analysis of the mRNA levels of *Nr2c2* and *Pparg*; n = 3 independent experiments. (c) Oil Red O staining; n = 5 independent experiments, scale bar = 50 μm. Data represent the mean ± SEM. Two-tailed student's t test was performed for (b). eWAT: epididymal white adipose tissue. HODEs: 9/13-HODEs.

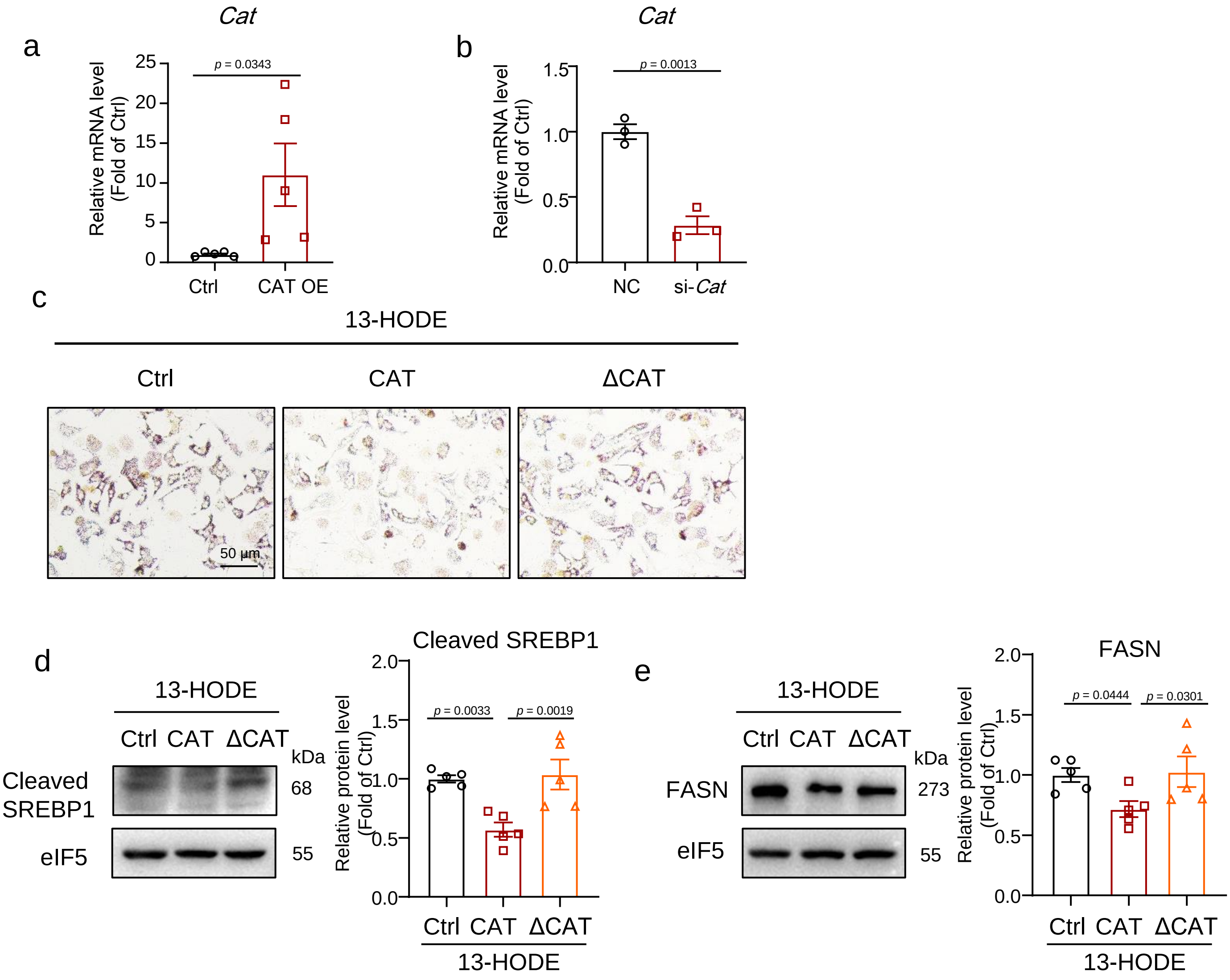

Fig. S11 mRNA level of *Cat* in CAT-overexpressing hepatocytes or Cat-knockdown hepatocytes.
Primary mouse hepatocytes were transfected with CAT-expressing plasmid (n = 5 independent experiments) **(a)** or si-*Cat* (n = 3 independent experiments) **(b)**: qPCR analysis of the mRNA level of *Cat*. **(c-e)** Primary mouse hepatocytes were transfected with CAT-expressing plasmid or plasmid expressing CAT lacking N-terminal arm (residues 5-70; ΔCAT) and treated with 13-HODE (1 μM) for 48 h: (c) Oil Red O staining; scale bar = 50 μm. Western blot analysis of protein levels of cleaved SREBP1 (d) and FASN (e); n = 3 independent experiments. Data represent the mean ± SEM. Two-tailed student's t test was performed for (a-b); One-way ANOVA with Fisher's LSD was performed for (d-e). CAT OE: catalase overexpression.

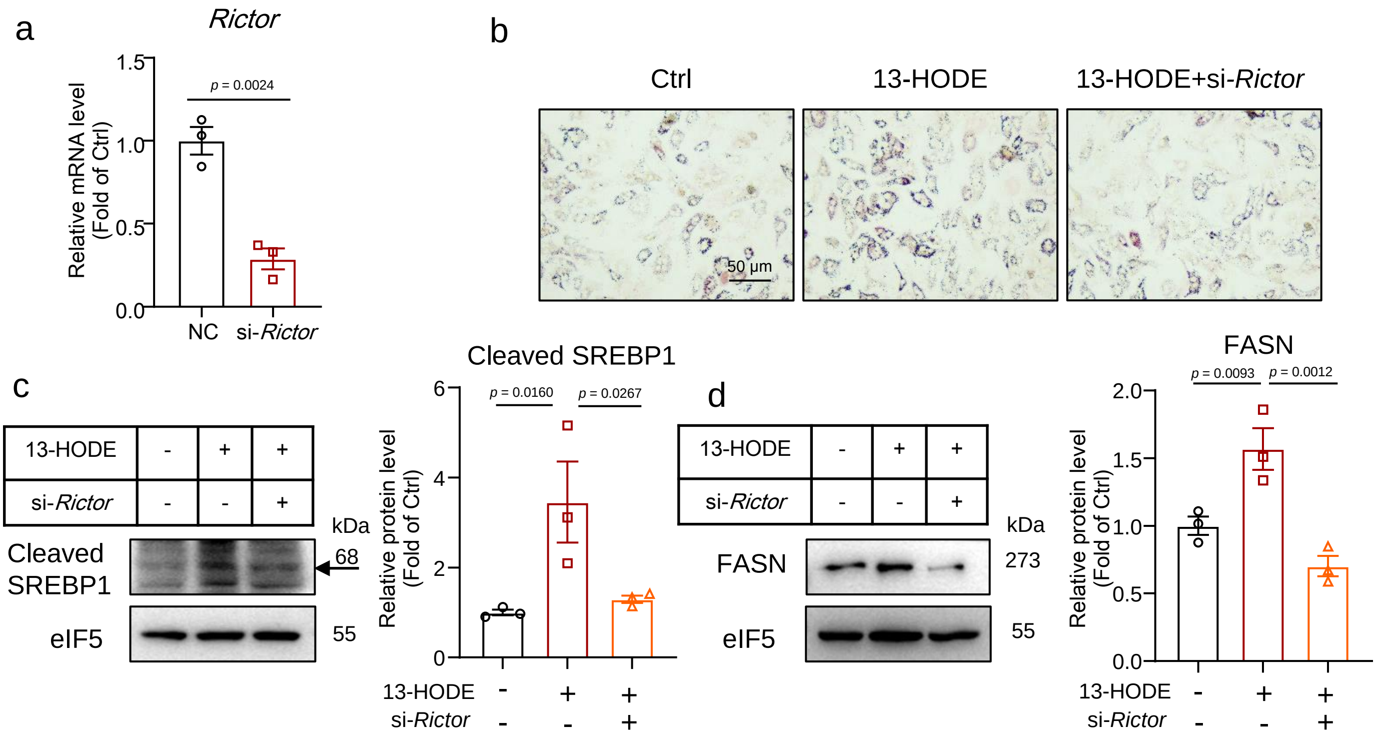

Fig. S12 Rictor knockdown suppressed 13-HODE induced hepatocyte steatosis. Primary mouse hepatocytes were transfected with si-*Rictor* with or without 13-HODE (1 μM) for 48 h: **(a)** qPCR analysis of the mRNA levels of *Rictor*. **(b)** Oil Red O staining; scale bar = 50 μm. **(c, d)** Western blot analysis of the protein levels of cleaved SREBP1 and FASN. n = 3 independent experiments. Data represent the mean ± SEM. Two-tailed student's t test was performed for (a); One-way ANOVA with Fisher's LSD was performed for (c-d).

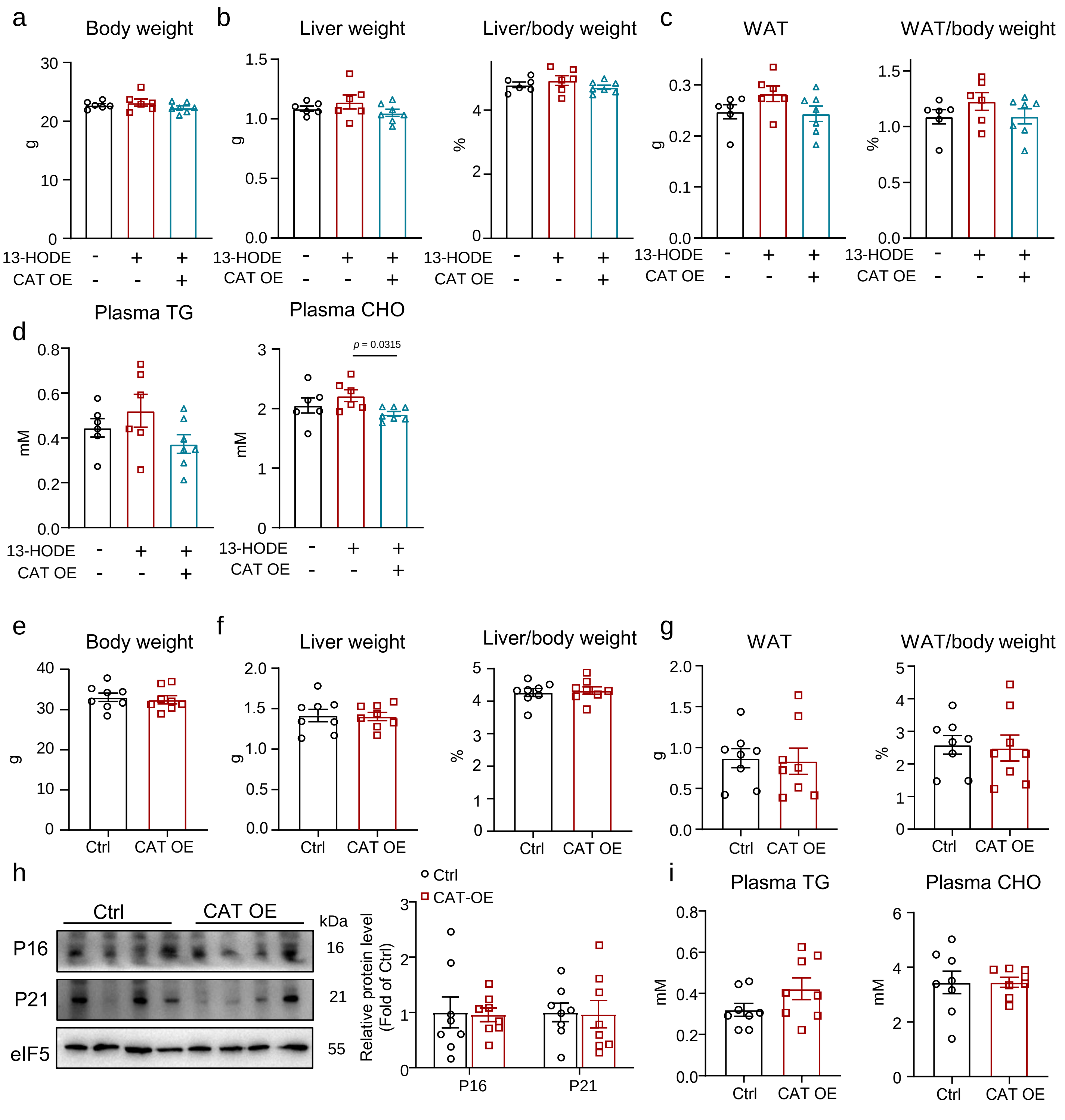

Fig. S13 Liver and WAT weights and plasma TG and CHO levels of 13-HODE treated and middle-aged mice with hepatocyte-specific overexpression of CAT. (a–d) Eight-week-old mice were injected with AAV–*Cat*–flag bearing TBG promoter or control AAV; 10 days after AAV injection, mice were treated with 13-HODE (0.5 μg/g body weight) or vehicle at a dose of once a day for 9 days: (a) body weight; (b) liver and liver/body weight; (c) epididymal WAT (eWAT) weight and eWAT/body weight; (d) plasma TG and CHO levels. n = 6 mice in Ctrl group and 13-HODE group; n = 7 mice in 13-HODE+CAT group. **(e–h)** Eight-month-old mice were injected with AAV–CAT–flag or control AAV; mice were sacrificed 2 months after AAV injection: (e) body weight; (f) liver and liver/body weight; (g) eWAT weight and eWAT/body weight; (h) western blot analysis of the protein levels of P16 and P21; (i) plasma TG and CHO levels. n = 8 mice per group. Data represent the mean ± SEM. One-way ANOVA with Fisher's LSD was performed for (a-b, right panel of c, d); Kruskal-Wallis test was performed for (left panel of c) and Two-tailed student's t test was performed for (e-i). TG: triglyceride; CHO: total cholesterol; CAT OE: catalase overexpression. eWAT: epididymal white adipose tissue.

Table S1. List of siRNA sequences used in cell experiments

si-RNA	Sense (5’-3’)	Anti-sense(5’-3’)
si-*Rictor (m)*	GCCAUCUGAAUAACUUCACUATT	UAGUGAAGUUAUUCAGAUGGCTT
si-*ALOX15 (h)*	GCUAUCAAAGACUCUCUAAAUTT	AUUUAGAGAGUCUUUGAUAGCTT
si-*Cat (m)*	CCCUCUUAUACCAGUUGGCAATT	UUGCCAACUGGUAUAAGAGGGTT
si-*Pparg (m)*	GAGAUCACAGAGUAUGCCAAATT	UUUGGCAUACUCUGUGAUCUCTT
si-*Nr2c2 (m)*	GCUCAUGAGCUCCAACAUATT	UAUGUUGGAGCUCAUGAGCTT
NC *(m/h)*	UUC UCC GAA CGU GUC ACG UTT	ACG UGA CAC GUU CGG AGA ATT

Mus musculus; h: Homo sapiens.

Table S2. List of oligonucleotide primer pairs used in quantitative RT-PCR and RT-PCR analysis

Target Gene	Sense (5'-3')	Anti-sense(5'-3')
Fasn (m)	TGGGTTCTAGCCAGCAGAGT	ACCACCAGAGACCGTTATGC
Thrsp (m)	GAGAACGACGCTGCTGAAA	GGGTAAGGATGTGATGGAGG
Cd36 (m)	TGGTCAAGCCAGCTAGAAA	CCCAGTCTCATTTAGCCAC
Cyp4a14 (m)	ATACGAGTCCTGCTTTATGACC	AACAGGAGCAAACCATAACCA
Elovl6 (m)	CTTTCCTGTTTTCTGCGCTGT	TGTTCATCAGATGCCGACCAC
Cat (m)	GAGGAAACGCCTGTGTGAGA	TAGTCAGGGTGGACGTCAGT
G6pc (m)	AGGAAGGATGGAGGAAGGAA	TGGAACCAGATGGGAAAGAG
Nr2c2 (m)	CATATTCACCACCTCGGACAAC	TGACGCCACAGACCACAC
Pparg (m)	TCTCCAGCATTTCTGCTCCA	TCAATGGCCATGAGGGAGTT
Srebp1a (m)	GGCCGAGATGTGCGAACT	TTGTTGATGAGCTGGAGCATG
Srebp1c (m)	GGCCATCGACTACATCCGCTTC	CGGGCTCAGAGTCACTACCAC
Gpr132 (m)	TACATCAGCACGGTGCCATT	AGGTGAAACGCAGGTAGTGG
IL1A (h))	AATCATCAAGCCTAGGTCAGC	CTTCATCTTGGGCAGTCACA
IL1B (h)	GTTGAAAGATGATAAGCCCACT	GTTATATCCTGGCCGCCTT
IL6 (h)	CACACAGACAGCCACTCACC	ATTTTCACCAGGCAAGTCTCC
CXCL8 (h)	CAGTTTTGCCAAGGAGTGCTA	TTTTCCTTGGGGTCCAGACA
β-actin (m)	CTGTCCCTGTATGCCTCT	ATGTCACGCACGATTTCC
18S (m/h)	GGAAGGGCACCACCAGGAGT	TGCAGCCCCGGACATCTAAG

Mus musculus; h: Homo sapiens.

Uncropped blots in Supplemental figures

Fig. S1b

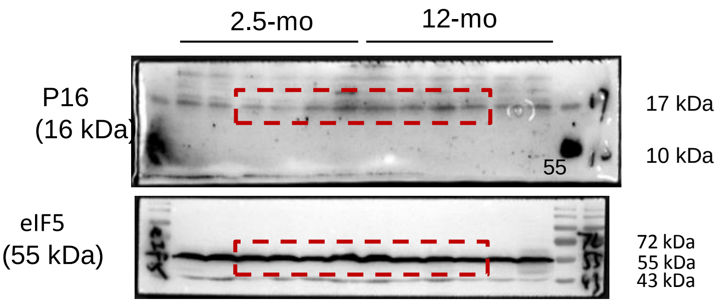

Fig. S1d

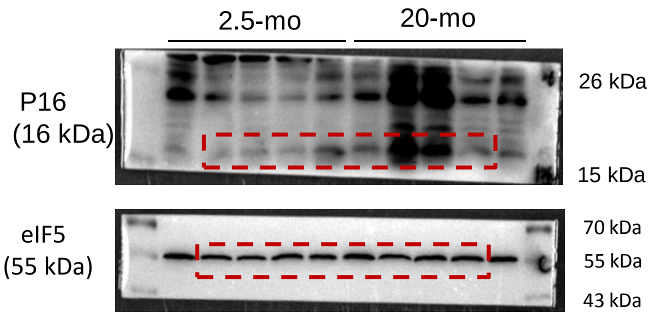

Fig. S2a

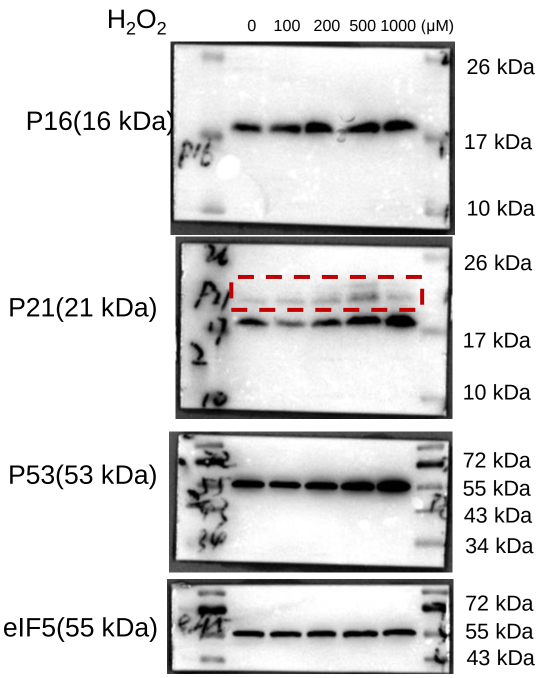

Fig. S5

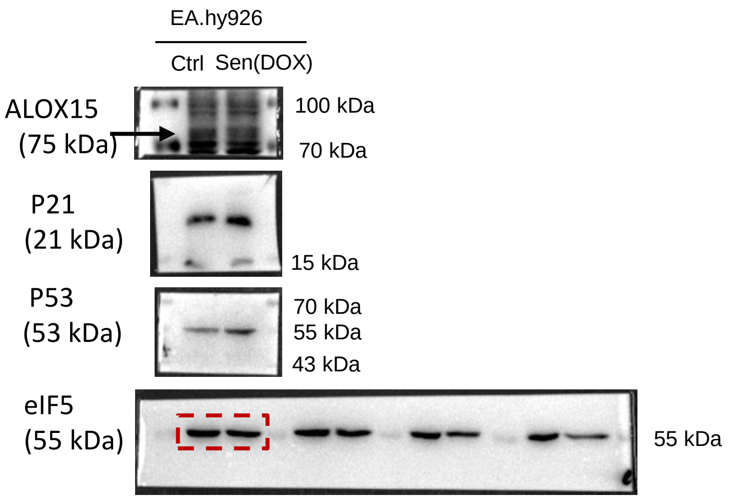

Fig. S6a

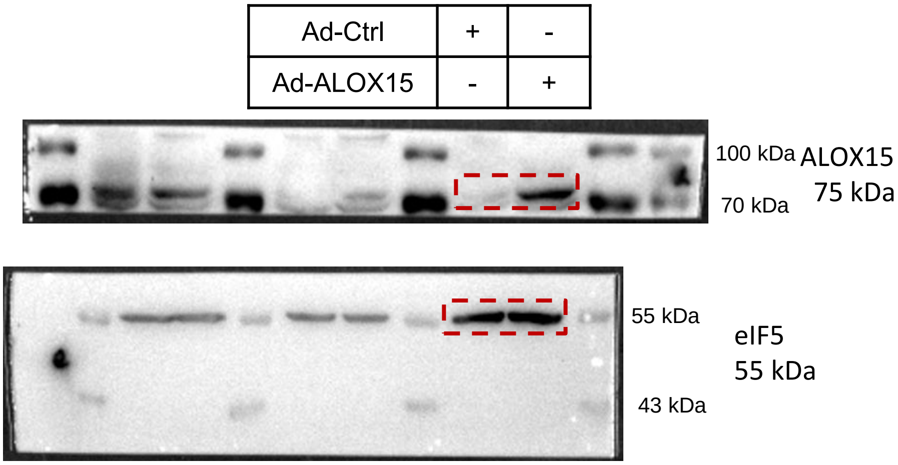

Fig. S6b

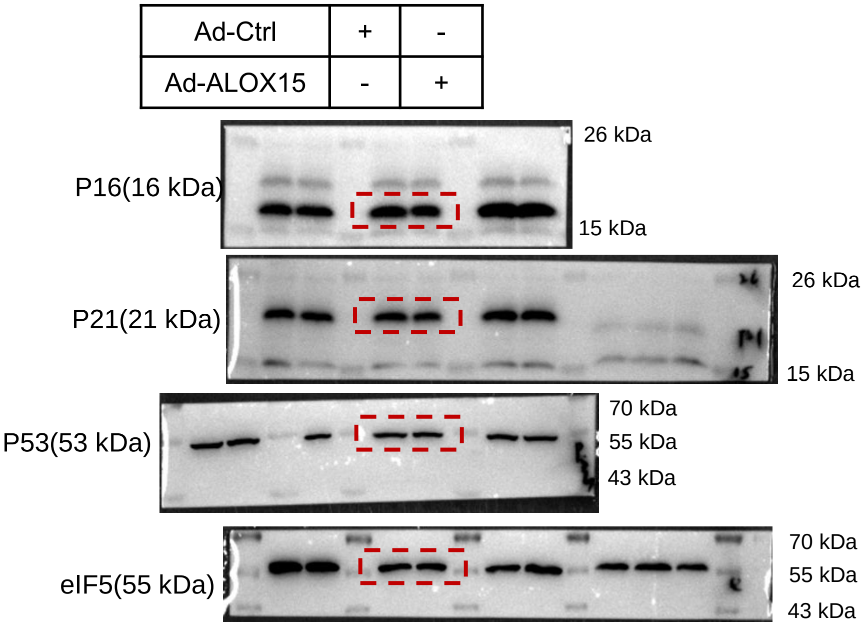

Fig. S7e

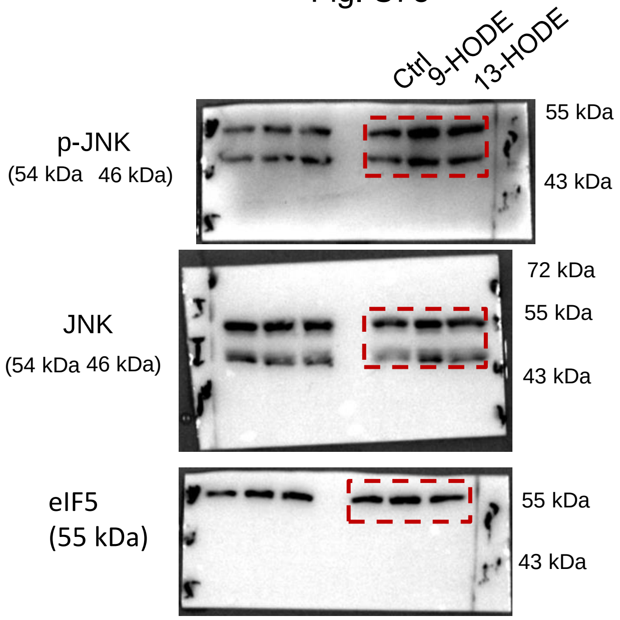
Ctrl
9-HODE
13-HODE
p-JNK
(54 kDa 46 kDa)
55 kDa
43 kDa
JNK
(54 kDa 46 kDa)
72 kDa
55 kDa
43 kDa
eIF5
(55 kDa)
55 kDa
43 kDa

Fig. S9a

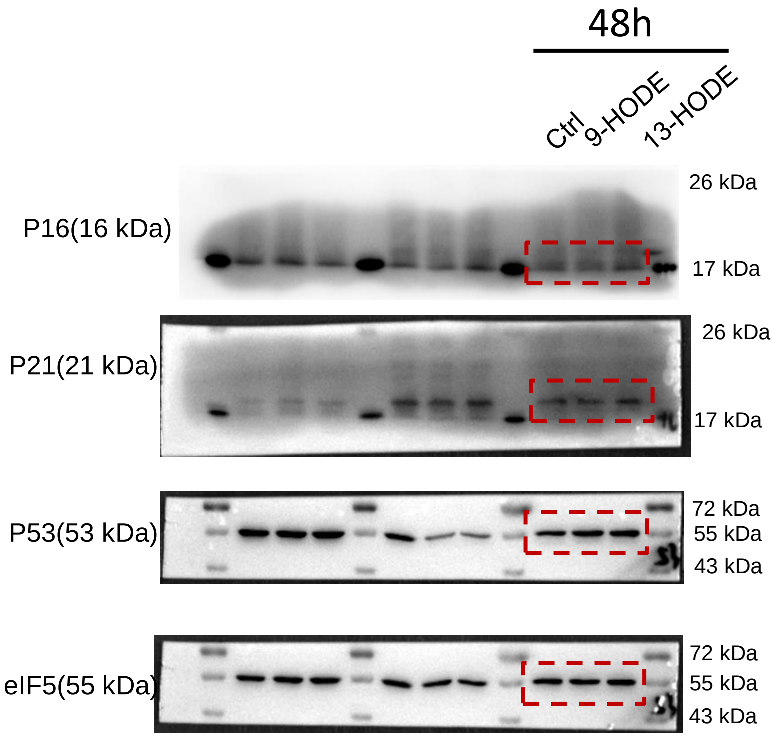

The PVDF membrane were incubated with anti-eiF5 antibody after stripping the P53 antibody.

Fig. S9b

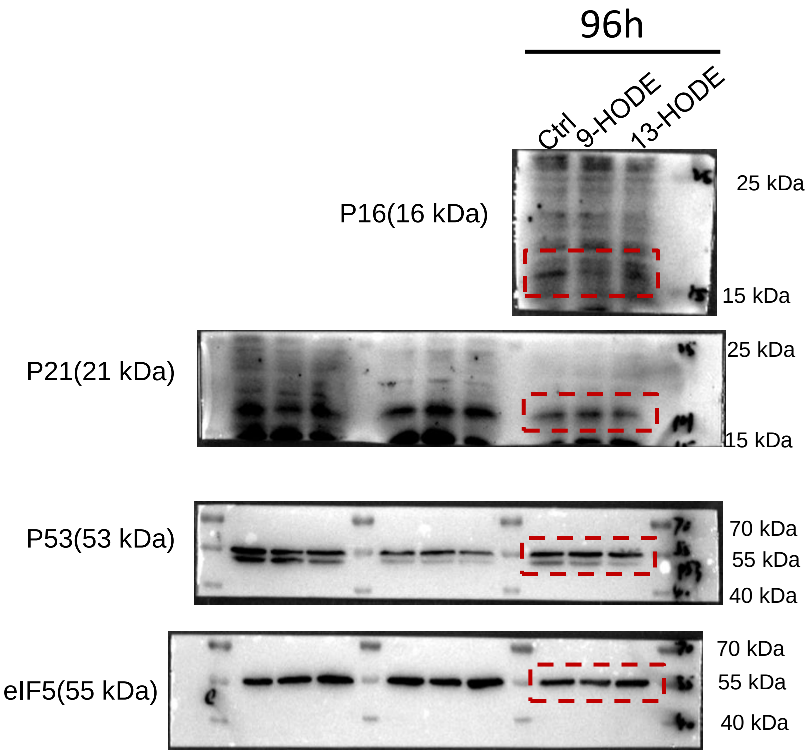

Fig. S11d

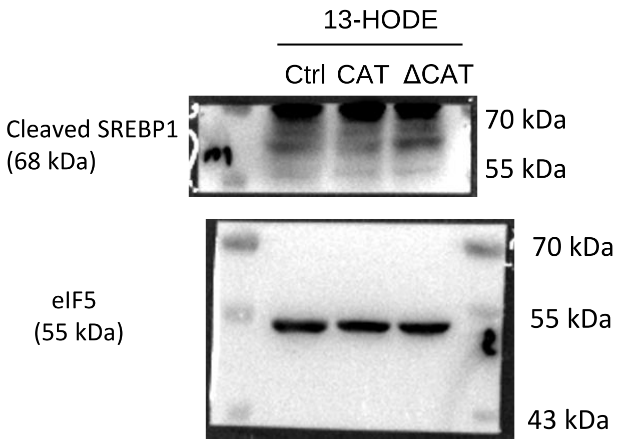

Fig. S11e

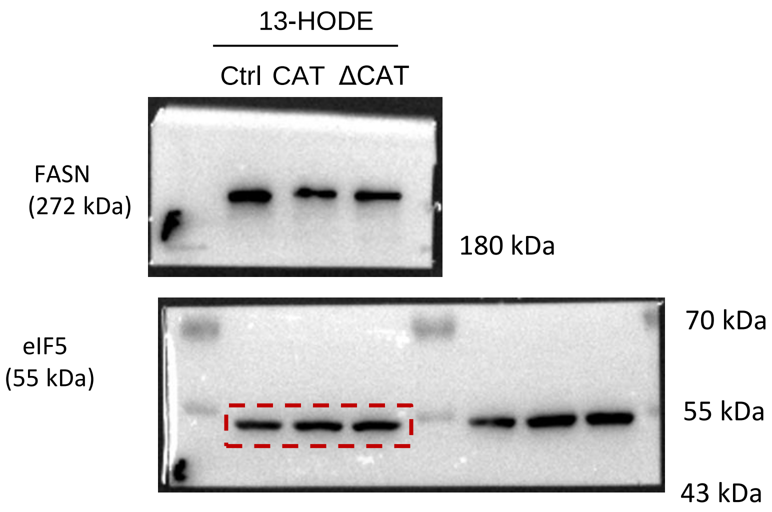

Fig. S12e

13-HODE	-	+	+
si-*Rictor*	-	-	+

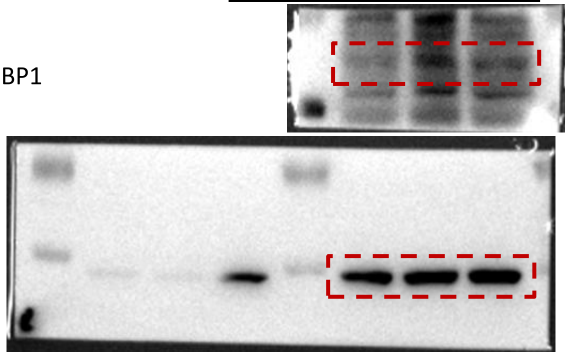

Fig. S12d

13-HODE	-	+	+
si-*Rictor*	-	-	+

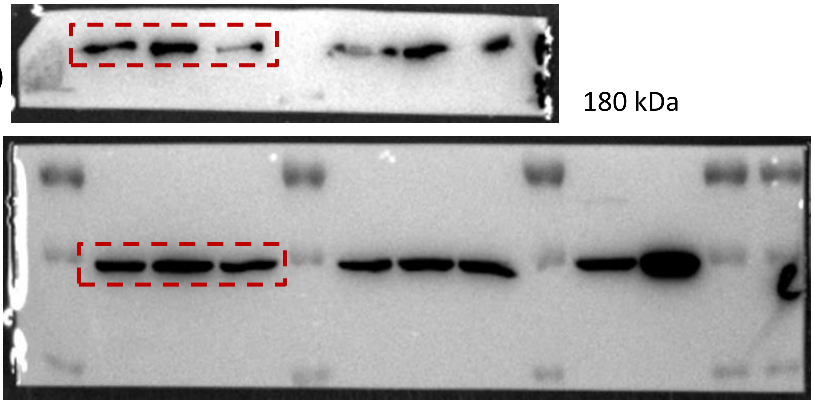

Fig. S13i

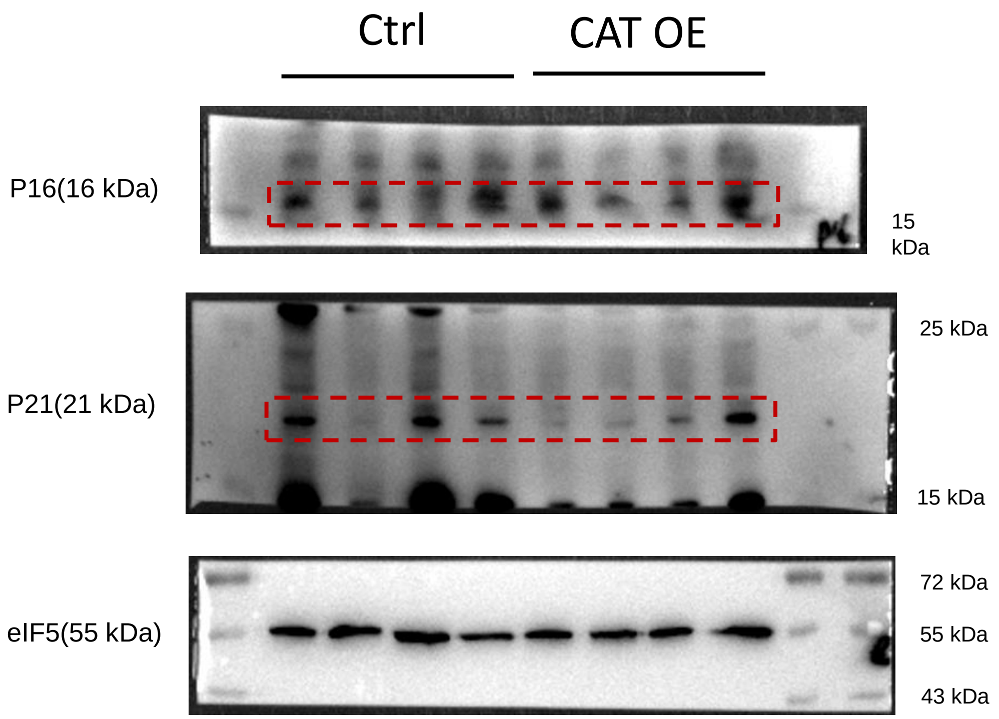
Ctrl
CAT OE
P16(16 kDa)
15 kDa
P21(21 kDa)
25 kDa
15 kDa
eIF5(55 kDa)
72 kDa
55 kDa
43 kDa
