# Supplementary material for: Senescence-associated 13-HODE production promotes age-related liver steatosis by directly inhibiting catalase activity
Source: Nat Commun. 2023 Dec 9;14:8151. doi: 10.1038/s41467-023-44026-z (PMC10710422; doi:10.1038/s41467-023-44026-z)
Supplement: Supplementary file 3 — Reporting Summary [file 41467_2023_44026_MOESM3_ESM.pdf]

nature portfolio

Corresponding author(s): Chunjiong Wang
Last updated by author(s): Nov 24, 2023

# Reporting Summary

Nature Portfolio wishes to improve the reproducibility of the work that we publish. This form provides structure for consistency and transparency in reporting. For further information on Nature Portfolio policies, see our Editorial Policies and the Editorial Policy Checklist.

## Statistics

For all statistical analyses, confirm that the following items are present in the figure legend, table legend, main text, or Methods section.

| n/a | Confirmed | |
|---|---|---|
| ☐ | ☒ | The exact sample size (*n*) for each experimental group/condition, given as a discrete number and unit of measurement |
| ☐ | ☒ | A statement on whether measurements were taken from distinct samples or whether the same sample was measured repeatedly |
| ☐ | ☒ | The statistical test(s) used AND whether they are one- or two-sided<br>*Only common tests should be described solely by name; describe more complex techniques in the Methods section.* |
| ☒ | ☐ | A description of all covariates tested |
| ☐ | ☒ | A description of any assumptions or corrections, such as tests of normality and adjustment for multiple comparisons |
| ☐ | ☒ | A full description of the statistical parameters including central tendency (e.g. means) or other basic estimates (e.g. regression coefficient) AND variation (e.g. standard deviation) or associated estimates of uncertainty (e.g. confidence intervals) |
| ☐ | ☒ | For null hypothesis testing, the test statistic (e.g. *F*, *t*, *r*) with confidence intervals, effect sizes, degrees of freedom and *P* value noted<br>*Give P values as exact values whenever suitable.* |
| ☒ | ☐ | For Bayesian analysis, information on the choice of priors and Markov chain Monte Carlo settings |
| ☒ | ☐ | For hierarchical and complex designs, identification of the appropriate level for tests and full reporting of outcomes |
| ☒ | ☐ | Estimates of effect sizes (e.g. Cohen's *d*, Pearson's *r*), indicating how they were calculated |

*Our web collection on statistics for biologists contains articles on many of the points above.*

## Software and code

Policy information about availability of computer code

| | |
|---|---|
| Data collection | 1. qPCR: QuantStudio 3 Real-Time PCR Systems<br>2.Western blot: Tanon 5200<br>3. Immunofluorescence: Olympus FV1200<br>4. Oil red and Immunohistochemistry:Nikon eclipse Ts2<br>5. ROS detection:Leica DFC3000 G<br>6. RNA-seq: Illumina Novaseq 6000<br>7. Targeted lipidomics : ABsciex 5500Q<br>8. Blood glucose : a glucometer monitor (Roche) |
| Data analysis | Graphpad Prism(8.0.2),Image J (1.47v),Image Pro PLUS(6.0.0.260) |

For manuscripts utilizing custom algorithms or software that are central to the research but not yet described in published literature, software must be made available to editors and reviewers. We strongly encourage code deposition in a community repository (e.g. GitHub). See the Nature Portfolio guidelines for submitting code & software for further information.

## Data

Policy information about availability of data

All manuscripts must include a data availability statement. This statement should provide the following information, where applicable:

- Accession codes, unique identifiers, or web links for publicly available datasets
- A description of any restrictions on data availability
- For clinical datasets or third party data, please ensure that the statement adheres to our policy

The raw RNA-sequence data of 9-HODE and 13-HODE treated mouse livers are available at the GEO datasets (accession number: GSE245002). The proteomics data of 13(S)-HODE binding protein has been submitted to PRIDE (PXD046933).The data used to generate the main results shown in the main figures and extended figures are available as source data, including uncropped western blots and lipidomics data.

## Research involving human participants, their data, or biological material

Policy information about studies with human participants or human data. See also policy information about sex, gender (identity/presentation), and sexual orientation and race, ethnicity and racism.

| | |
|---|---|
| Reporting on sex and gender | Not applicable. |
| Reporting on race, ethnicity, or other socially relevant groupings | Not applicable. |
| Population characteristics | Not applicable. |
| Recruitment | Not applicable. |
| Ethics oversight | Not applicable. |

Note that full information on the approval of the study protocol must also be provided in the manuscript.

# Field-specific reporting

Please select the one below that is the best fit for your research. If you are not sure, read the appropriate sections before making your selection.

☒ Life sciences ☐ Behavioural & social sciences ☐ Ecological, evolutionary & environmental sciences

For a reference copy of the document with all sections, see nature.com/documents/nr-reporting-summary-flat.pdf

# Life sciences study design

All studies must disclose on these points even when the disclosure is negative.

| | |
|---|---|
| Sample size | Sample size was determined on experiment feasibility and material availability. |
| Data exclusions | No data was excluded. |
| Replication | Data show the means ± Standard Error of the Mean (SEM) of at least three biological replicates with the n indicated in each experiment. |
| Randomization | Animals and cell samples were randomly assigned to treatment groups. |
| Blinding | The treatments provided to mice and cell were not blinded for practical reasons to avoid mix up. To prevent bias, the investigators were blinded to group allocation during data collection and analysis. |

# Reporting for specific materials, systems and methods

We require information from authors about some types of materials, experimental systems and methods used in many studies. Here, indicate whether each material, system or method listed is relevant to your study. If you are not sure if a list item applies to your research, read the appropriate section before selecting a response.

## Materials & experimental systems

| n/a | Involved in the study |
|---|---|
| [ ] | [x] Antibodies |
| [ ] | [x] Eukaryotic cell lines |
| [x] | [ ] Palaeontology and archaeology |
| [ ] | [x] Animals and other organisms |
| [x] | [ ] Clinical data |
| [x] | [ ] Dual use research of concern |
| [x] | [ ] Plants |

## Methods

| n/a | Involved in the study |
|---|---|
| [x] | [ ] ChIP-seq |
| [x] | [ ] Flow cytometry |
| [x] | [ ] MRI-based neuroimaging |

# Antibodies

Antibodies used

Primary antibodies for western blot analysis:anti-p16INK4(10883-1-AP, Proteintech, Wuhan, China; dilution 1:1000), anti-p21 (10355-1-AP, Proteintech, Wuhan, China; dilution 1:1000), anti-p53(1C12) (#2524, cell signaling technology, Danvers, MA, USA; dilution 1:1000), anti-eIF5(E-10) (sc-28309, santa cruz, Dallas, TX, USA; dilution 1:5000), anti-FASN(10624-1-AP, Proteintech, Wuhan, China; dilution 1:1000), anti-SREBF1(14088-1-AP, Proteintech, Wuhan, China; dilution 1:1000), anti-catalase(21260-1-AP, Proteintech, Wuhan, China; dilution 1:1000), anti-flag(#14793, cell signaling technologyDanvers, MA, USA; dilution 1:1000),anti-15 lipoxygenase 1(ab244205, abcam, Cambridge, UK; dilution 1:1000).
Second antibodies for western blot analysis:Goat Anti-Mouse IgG(BL001A,biosharp,Anhui,China;dilution 1:5000),Goat Anti-Rabbit IgG (BL003A,biosharp,Anhui,China;dilution 1:5000).

Primary antibodies for immunofluorescence:anti-γH2AX (phospho S139) antibody (ab81299, Abcam, Cambridge,UK;dilution 1:100),anti-15-LO antibody (sc-133085, Santa Cruz, Dallas, TX;dilution 1:50),anti-p16INK4(10883-1-AP, Proteintech, Wuhan, China; dilution 1:50).
Second antibodies for immunofluorescence:Goat Anti-Rabbit IgG H&L (Alexa Fluor® 488) (ZF-0511, ZSGBBIO, Beijing, China, 1:200 dilution),Goat Anti-Mouse IgG H&L (Alexa Fluor® 488) (ZF-0512, ZSGBBIO, Beijing, China, 1:200 dilution),Goat Anti-Rabbit IgG H&L (Alexa Fluor® 594)(ZF-0516, ZSGBBIO, Beijing, China, 1:200 dilution).

Primary antibody for immunohistochemical:anti-F4/80 (#70076, Cell Signaling Technology, Danvers, MA, USA; dilution1:200 ).
Second antibody for immunohistochemical:HRP-labeled Goat Anti-Rabbit IgG(H+L) (A0208, Beyotime, Shanghai, China, dilution1:200 ).

Validation

Antibodies have been validated by company and detailed information could be found on the manufactures' websites .

anti-p16INK4(10883-1-AP, Proteintech, Wuhan, China)
https://www.ptglab.com/Products/P16,P19-Antibody-10883-1-AP.htm

anti-p21(10355-1-AP, Proteintech, Wuhan, China)
https://www.ptglab.com/products/P21-Antibody-10355-1-AP.htm

anti-p53(1C12) (#2524, cell signaling technology, Danvers, MA, USA)
https://www.cellsignal.cn/products/primary-antibodies/p53-1c12-mouse-mab/2524?site-search-type=Products&N=4294956287&Ntt=%232524&fromPage=plp&_requestid=6584207

anti-eIF5(E-10) (sc-28309, santa cruz, Dallas, TX, USA)
https://www.scbt.com/p/eif5-antibody-e-10?requestFrom=search

anti-FASN(10624-1-AP, Proteintech, Wuhan, China)
https://www.ptglab.com/products/FASN-Antibody-10624-2-AP.htm

anti-SREBF1(14088-1-AP, Proteintech, Wuhan, China)
https://www.ptglab.com/products/SREBF1-Antibody-14088-1-AP.htm

anti-catalase(21260-1-AP, Proteintech, Wuhan, China)
https://www.ptglab.com/products/CAT-Antibody-21260-1-AP.htm

anti-flag(#14793, cell signaling technologyDanvers, MA, USA)
https://www.cellsignal.cn/products/primary-antibodies/dykddddk-tag-d6w5b-rabbit-mab-binds-to-same-epitope-as-sigma-aldrich-anti-flag-m2-antibody/14793?site-search-type=Products&N=4294956287&Ntt=%2314793&fromPage=plp&_requestid=6585813

anti-15 lipoxygenase 1(ab244205, abcam, Cambridge, UK)
https://www.abcam.cn/products/primary-antibodies/15-lipoxygenase-1-antibody-epr22138-ab244205.html

anti-γH2AX (phospho S139) antibody (ab81299, Abcam, Cambridge,UK)
https://www.abcam.cn/products/primary-antibodies/gamma-h2ax-phospho-s139-antibody-ep8542y-ab81299.html

anti-15-LO antibody (sc-133085, Santa Cruz, Dallas, TX,USA)
https://www.scbt.com/p/15-lo-antibody-b-7?requestFrom=search

anti-F4/80 (#70076, Cell Signaling Technology, Danvers, MA, USA)
https://www.cellsignal.com/products/primary-antibodies/f4-80-d2s9r-xp-rabbit-mab/70076

## Eukaryotic cell lines

Policy information about cell lines and Sex and Gender in Research

| | |
|---|---|
| Cell line source(s) | HepG2 (ATCC), Raw264.7 (ATCC), EA.hy926(Cell Bank/Stem cell bank of Chinese Academy of Sciences) |
| Authentication | None of the cell lines were authenticated |
| Mycoplasma contamination | All cell lines were tested negative for mycoplasma contamination. |
| Commonly misidentified lines (See ICLAC register) | No commonly misidentified cell lines were used. |

## Animals and other research organisms

Policy information about studies involving animals; ARRIVE guidelines recommended for reporting animal research, and Sex and Gender in Research

Laboratory animals

All protocols and animal studies were performed in accordance with the Guide for the Care and Use of Laboratory Animals by the US National Institutes of Health (NIH Publication No. 85–23, updated 2011). In addition, the Laboratory Animal Management and Use Committee of Tianjin Medical University, Tianjin, China, approved this study. All mice were housed in a temperature-controlled environment of 22-23°C, 40–70% humidity in individually ventilated cages with wood pieces as bedding with 12 h light/dark cycles and received food and water ad libitum, except for fasting experiments. If not indicated, mice were fed with chow diet (1010001, Jiangsu Xietong Pharmaceutical Bio-engineeringXietong Shengwu, Jiangsu).

Male C57BL/6 mice were obtained from SPF (Beijing) Biotechnology Co., Ltd. Mice aged 2.5-, 12-, and 20-month-old were used to study age-related liver steatosis. In addition, to study the effect of 9/13-HODEs on hepatic steatosis, 8-week-old mice were intraperitoneally injected with a combination of 9-HODE (Item No. 38400, Cayman Chemical, Ann Arbor, MI) and 13-HODE (Item No. 38600, Cayman Chemical) [equal amounts of 9-HODE and 13-HODE with a combined dose of 0.5 μg/g body weight] every day for 9 days. The mixture of 9-HODE and 13-HODE was dissolved in a mixture of PEG400 and water (1:5) to a final concentration of 0.1 mg/mL. The control mice were intraperitoneally injected with the solvent.

To specifically overexpress CAT in hepatocytes, 8-month-old mice were injected with AAV8-Cat (mouse)-flag with the TBG promoter (1.5 × 1011 vg/mouse; GeneChem Co., Ltd., Shanghai), and the mice were sacrificed 2 months later.

To investigate the effects of CAT on 13-HODE-induced liver steatosis, 8-week-old mice were injected with AAV-Cat-flag with the TBG promoter for 10 days, before being administered 13-HODE (0.5 μg/g body weight) intraperitoneally every day for 9 days before sacrifice.

For HFD-fed mice, 8-week-old male mice were fed a 45% HFD (Medicience, Yangzhou) or chow diet for 12 weeks to induce liver steatosis.

Mice were euthanized by exsanguination after being anaesthetized with tribromoethanol (0.24mg/g body weight).

| | |
|---|---|
| Wild animals | No wild animals were involved in this study. |
| Reporting on sex | Sex was not considered a critical factor in this study. Based on the 3R principle of animal experiments, we used male mice as representative in this study. |
| Field-collected samples | No field collected samples were used in this study. |
| Ethics oversight | All animal experiments were approved by the Laboratory Animal Management and Use Committee of Tianjin Medical University (Document NO. TMUaMEC 2022012). |

Note that full information on the approval of the study protocol must also be provided in the manuscript.
